# Editorial: Investigating Human Nature and Communication through Robots

**DOI:** 10.3389/fpsyg.2016.01784

**Published:** 2016-11-18

**Authors:** Shuichi Nishio, Hideyuki Nakanishi, Tsutomu Fujinami

**Affiliations:** ^1^Hiroshi Ishiguro Laboratory, Advanced Telecommunications Research Institute InternationalKyoto, Japan; ^2^Department of Adaptive Machine Systems, Osaka UniversityOsaka, Japan; ^3^School of Knowledge Science, Japan Advanced Institute of Science and TechnologyIshikawa, Japan

**Keywords:** robot, communication, enhancement, human nature, teleoperation, embodiment

The aim of this research topic was to gather findings and hypothesis on how robotic devices have changed, or may change, ways of communication between people. In the last two decades, people acquired new means for communication; cellphones, e-mail, chat, SNS, and so on. With such communication media, along with the progress in information technologies and devices such as World Wide Web (WWW) and smartphones, ones lifestyle has rapidly changed. We can now talk with others anywhere and anytime, can send and receive not just text or voice but also images, movies to express our ideas and feelings in finer detail. Such changes not only increased the bandwidth and relaxed the distance limitation of communication; they also changed how people communicate with each other. Such changes provided researchers with new sources and methods for investigating human nature such as cognitive properties and sociological tendencies.

Now various types of robots that are aimed to work in our daily environment are developed and starting to appear in markets. Some robots can make simple conversation with people autonomously. Some cannot speak but people anthropomorphize them and talk to them. Some work as a mobile video chat system. Robots differ from existing information devices in that they can physically interact with real world objects. They can move round in the world we live, can carry things, can touch people or can be touched by people. You can feel a strong presence of the robot. Having conversation with such robots, or having conversation with other persons through such robots may re-define the meaning of communication.

People are starting to apply this new possibility in various fields. Some are making theater performance and art works with robots. Some are trying to use robots as means to understand and to talk to people with cognitive impairments such as dementia and autism. And some are using robots to refine communication with others. Such trials, as well as efforts to refine robots so that people can easily interact with them, are shedding lights on previously unknown human nature; e.g., how we recognize ourselves and others, what it is to have communication with others.

In this research topic, human communication with robots or through robots were examined from versatile aspects. Two papers examined how robots or their behavior are recognized by people. Matsuda et al. tested if infant can discriminate androids, a robot with very humanlike appearance. It is well known that people tend to feel “uncanniness” toward androids (MacDorman and Ishiguro, 2006). They examined if this uncanny feeling is equipped in people from birth or is developed during growth. Bremner and Leonards examined whether human can process gestures produced by robots in the same way as produced by others humans. When we speak to others, non-verbal elements are generated inevitably due to our body and such elements are processed in combination to speech. The question is, will this multi-modal processing be triggered for robots as well.

Two papers examined how robots can be made to affect people. Tanaka et al. tested how various factors in a robot, especially its physical embodiment, affect its social telepresence. That is, if robot that have physical bodies are better than telephones or video phones or not, in what way, and how robots can be used to make the effect stronger. Hori et al. tried to express emotion with robots, not by using body motions or facial expressions, but by changing illumination patters.

Four papers tested how communicating through robots would affect people. Damholdt et al. how elderly citizens will respond to a teleoperated robot. That is, when having a conversation through a robot that is controlled by another person, what personal factors of the elderly citizen will affect their attitude toward the robot. Kuwamura et al. focused on elderlies with dementia. They performed a long term testing in a care facility using a teleoperated robot, and checked how the robot is accepted and how people interacted with the robot. Yamazaki et al. examined if having a conversation through a robot, instead of using a telephone, would reduce stress. Nakanishi et al. describes their attempt to use a teleoperated robot as teaching tool in school. By using a huggable device, they examined if talking to children through the device would help the children to concentrate more on teacher.

And finally, Corti and Gillespie showed a unique setup on “robotic” teleoperation. Instead of having an operator person who controlls a robot, they used an artificial chat system (chat bot) to determine what to speak in conversation with others. This “Echoborg” is used to perform several testings and the authors also discuss on the possibility of creating an androids that speak autonomously.

## Author contributions

SN wrote the article; HN and TF provided comments on the draft.

## Funding

Part of this work has been supported by: JST/ERATO; Grants-in-Aid for Scientific Research 25220004; Strategic Platforms for Innovation and Research.

### Conflict of interest statement

The authors declare that the research was conducted in the absence of any commercial or financial relationships that could be construed as a potential conflict of interest.
